# Can Desire and Wellbeing Be Promoted in Adolescents and Young Adults Affected by Cancer? PhotoTherapy as a Mirror That Increases Resilience

**DOI:** 10.3389/fpsyg.2020.00966

**Published:** 2020-05-12

**Authors:** Ines Testoni, Elena Tomasella, Sara Pompele, Maurizio Mascarin, Michael Alexander Wieser

**Affiliations:** ^1^Department of Philosophy, Sociology, Education and Applied Psychology (FISPPA), University of Padua, Padua, Italy; ^2^Oncological Reference Center (CRO), Aviano, Italy; ^3^Institute of Psychology, University of Klagenfurt, Klagenfurt, Austria

**Keywords:** PhotoTherapy, wellbeing, desire, cancer, adolescents, young adults, qualitative research

## Abstract

PhotoTherapy represents a recent psychotherapeutic intervention which, through the medium of photography, has the objective of helping a patient reach a higher self-knowledge and explore thoughts, desires, and intimate perceptions that would otherwise remain hidden. Because of this, it may help people overcome their cognitive and emotional boundaries and bring out their interiority, even when exploring some of the most distressing themes. The objective of the present research was to demonstrate that the elaboration and expression of inner desires, fears and needs of wellbeing among adolescents and young adults, who were either sick or had been cured of cancer, could be facilitated through the use of PhotoTherapy. Their responses were also compared with those of healthy young adults, in order to be able to explore the kind of impact such a pathology can have on those who are affected by it and highlight eventual differences in the kind of desires and needs expressed instead by those who never had a diagnosis of cancer. A qualitative research design was adopted. 45 people took part in the study, aged between 12 and 25 (mean age 19). The participants were divided into three groups: those currently being treated for cancer, those with a history of cancer and those who had no experience of cancer (third group). From the data analysis, different preferences and attitudes toward the presented pictures and themes emerged among the participants, depending on the specific group they belonged to. The article discusses these preferences and considers how PhotoTherapy can help treat psychological distress caused by cancer.

## Introduction

PhotoTherapy is a recent form of art therapy utilized in both the therapeutic area and supportive interventions aimed to promote wellbeing by facilitating people’s adjustments to stressful events and relieving psychological pain (Stewart, 1979). It utilizes the medium of photography, either directly produced by participants or collected from external sources. The main objective of the reflection on images is that of helping people to reach a higher self-knowledge and to explore states of mind, thoughts, desires, and intimate perceptions that would otherwise remain hidden, to the point that Joel Walker (1982), one of the fundamental pioneers in the conceptualization and implementation of PhotoTherapy, describes it as *“a catalyst in psychotherapy”* (p. 454).

Judy Weiser (1999, 2010), another theorist of this methodology, describes five forms of PhotoTherapy, which are mutually interconnected but nonetheless substantially different from each other: elaborating on pictures made by the patients; elaborating on photos of patients taken by other people; producing self-portraits; reflecting on family albums; and engaging in what are known as “photo-projective” interactions. The photo-projective form uses pictures chosen by the therapist to open the dialogue. The images are considered metaphors of the individual’s internal world. This form is particularly important, as the psychological projection characterizes all the other strategies, and can be considered the basic psychological dimension activated in PhotoTherapy (Weiser, 2004). In this peculiar process, people spontaneously express their inner meanings, transforming the photos into a mirror that reflects their most significant personal experiences and feelings (Musacchi, 2016), allowing them to achieve a meaningful moment of self-confrontation, as highlighted by phototherapist Fryrear (1983). To activate this dynamic, the therapist does not need to directly address significant and potentially distressing themes but simply asks patients how they interpret the images and what kind of thoughts, feelings and memories emerge.

This dynamic allows participants to confront strong topics and emotions while maintaining a protective distance (Weiser, 1999), and it helps the therapist explore not only patients’ discomfort but also positive aspects, moving the therapeutic process toward wellbeing (Weiser, 1990). All these characteristics make use of photographs, which are particularly useful for facing threatening issues such as psychological and physical illness. Walker (1991) has also successfully applied PhotoTherapy with terminally ill people, thus highlighting how this methodology can represent a useful tool even in those cases in which the extremely delicate theme of human fragility and death needs to be addressed.

Indeed, this technique has been successfully applied in a variety of situations; for example, with HIV-positive people (Weiser, 1999), with adolescents presenting socio-relational difficulties (Milford et al., 1983), or in the psychosocial field, with the objective of facilitating integration, and contrasting the marginalization of specific social groups (Musacchi, 2016).

In particular, it has already been shown to be efficacious with young people, enabling them to manage issues inherent in illness and death (Testoni et al., 2018a) through activities which help them face future crucial moments (Bell et al., 2009).

Art Therapist Moon (1998) who frequently worked with adolescents, in this regard highlighted how PhotoTherapy could represent a valuable alternative to more traditional kinds of psychotherapy, since, in his experience, these patients do not usually feel at ease with a merely verbal approach, while, on the other hand, many adolescents find it easier to express their complex inner world through the arts, since *“for adolescents in need of psychotherapy, art is not a frill or a time-filler, but rather, a dynamic, validating, integrating, expressive and entirely natural and necessary activity”* (1998, p. 12).

Consequently, this technique becomes even more important with seriously ill young people, since there is also the necessity to help them make sense of their sickness and improve their wellbeing (Testoni, 2016), which still appears to be rather challenging (Jones, 2012; Smith et al., 2013; Sawyer et al., 2017).

Literature considering the efficacy of PhotoTherapy with young people affected by cancer is scarce. Among the rare studies is that of Gaggiotti et al. (2019), which showed the positive effect of the production of pictures with 27 adolescents and young adults suffering from the illness. Other projects used photography as a means to facilitate young people’s reflection upon the theme of death (Testoni et al., 2019b, e). These projects involved the production of photos and confirmed how the procedure significantly facilitated a more serene discussion of difficult issues.

## Materials and Methods

### Objectives

The objective of the present qualitative study was to explore how PhotoTherapy can facilitate the elaboration and expression of inner desires and needs for the wellbeing of adolescents and young adults in relation to their experience of cancer. The results were compared to those of peers who had never faced such a diagnosis, in order to be able to explore the kind of impact such a pathology can have on those who are affected by it and highlight eventual differences in the kind of desires and needs expressed instead by those who never had a diagnosis of cancer.

### Participants

Forty-five adolescents and young adults took part in the study. The participants were divided into three groups based on their health conditions: those currently being treated for cancer; those in the follow-up phase of a history of cancer (within the last 5 years); and those with no experience of oncological or other severe pathologies. The inclusion criteria were as follows: understanding and speaking Italian well; having no psychiatric illness; seeing well; and being motivated to participate in the study. Table 1 describes the composition of the three groups.

**TABLE 1:** Participants’ characteristics.

Participants’ group	Age		Gender	
	Mean	Standard deviation	Males	Females
Group n. 1	22	4.64	12	3
Group n. 2	18.87	3.38	6	9
Group n. 3	17.87	4.05	5	10
All participants	19.58	4.34	23	22

Participants in the first group were under antineoplastic treatment at an oncological center in Northern Italy which has an area that is specialized in treatment and multidisciplinary support for young patients. The second group was composed of people who had terminated their treatment for cancer at the same hospital within the past 5 years. Both groups were recruited thanks to the oncologist working with youth in the same department. The PhotoTherapy was administered in a specific room of the hospital (the “Room of the Self”) by a psychologist trained in Weiser’s methodology. When participants were not well, the procedure was conducted in their rooms. Participants falling into the third group were recruited from local high schools and universities. An announcement describing the objectives and main procedures of the research was attached to message boards at the different institutions.

### Data Collection

The intervention used the photo-projective method elaborated by Weiser (1990, 2004). Participants were invited to choose the picture from among a group of 35 photos that had previously selected by the researchers and that could most adequately facilitate a reflection upon the theme of desire. The pictures were divided into six clusters of meaning: leisure activities, sports and arts (e.g., pictures of books, promenades, and sports, etc.); medical treatments (e.g., tightrope walker, healthcare professionals, etc.); school and working career (e.g., classroom, office, etc.); family life (e.g., breakfast, family happily running on a lawn, etc.); personal identity (e.g., a girl sitting in dim light; a running man, etc.); and change (e.g., some gears, a girl walking on railway tracks, etc.). As suggested by Weiser (2010), the consistent number of photographs selected (35) was aimed to offer the greatest variety of pictures possible, to help participants find a theme, a representation or an element that could attract their attention. In this regard, there were ten pictures which more explicitly referred to common everyday activities and themes (for example the picture of some different sports in the cluster n. 1 “Leisure activities, sports and arts”), however, the rest of them were purposely more ambiguous, in order to stimulate the participants’ creativity and to allow them to pour their inner world in them, giving their unique interpretation of the photograph and making it a symbol of their personal thoughts, emotions, desires, and fears (for example the picture representing some gears from cluster n.6 “Change”).

All pictures were colored, some with brighter, highly saturated tints, and others with darker shades. In 15 pictures there were people represented, both during some common activities (for example a family having a walk on a beautiful field), and in a more complex, symbolical condition (for example a girl crying sitting on the floor, a tightrope walker), while in the remaining ones only objects or landscapes were visible. All the pictures were in focus, with a high resolution.

Some examples of the pictures used for the present research, and more precisely the very pictures chosen by the participants direclty quoted in the “Results” section of the manuscript, can be found as Supplementary Material for the present article.

Each participant took part in one encounter. At first they were asked to look at the 35 pictures displayed in front of them, and to freely choose, without rushing, the ones that appealed to them the most, and which reminded them of their strongest, deepest desire and related emotions, thoughts and needs. This first request had the aim to allow the participants to take their time, explore the pictures, and start to think about their personal feelings and desires. In doing this, the participants could move, touch and pick up all the pictures they wanted, if they felt the need.

After that, as a second step, the participants were asked to select only one of the previously chosen pictures, the one which appeared to be more meaningful to them.

Subsequently, the interview could start, and as a first question they were asked to try and define desire, in their own words (i.e., “In your opinion, what is desire?”), which was useful for finding a starting point for a dialogue with the PhotoTherapist on needs, hopes for the future and wellbeing.

The participants were then asked to explain why they had chosen that final, peculiar picture, what it meant to them and what kind of feelings, thoughts and memories it enhanced in them.

Moving from the description of the chosen picture and of the specific effect it had on the participants, the researcher then proceeded to further explore the participants’ needs and desires, together with their eventual fears and uncertainties concerning their present condition and their future.

Since the interview was a semi-structured one, even though the PhotoTherapist followed a basic pattern of questions for all the participants (starting from a first broader question concerning their definition of desire, asking subsequently about the chosen picture and then progressively exploring the related thoughts, needs, fears and hopes of each person), the dialogue was not conducted in a strict way but followed instead the participants’ spontaneous rhythm and interests, while the PhotoTherapist simply directed the flow of the conversation, in order to offer each participant the possibility to fully express his/her opinions and feelings, and to introduce different themes, provided that they were related to the picture he/she chose, and to the broader ideas of personal desires, needs, and fears.

At the end of each meeting, the researcher also gathered feedback from each participant concerning the PhotoTherapy session, through the simple questions: “What do you think of this activity, did you like it? Is there anything you think should be improved?”

This allowed the participants to express their point of view concerning the experience, and to eventually suggest any possible improvements.

All the participants answered enthusiastically, reporting high levels of satisfaction concerning the PhotoTherapy experience.

### Data Analysis

The research part of the project adopted a qualitative design (Denzin and Lincoln, 2005) and analyzed participants’ narratives, which had been audio-recorded and literally transcribed. The corpus obtained was analyzed using thematic analysis, which allowed texts to be examined in terms of their principal contents (Testoni et al., 2019d). The analysis proceeded by recognizing the reasons that had led participants to choose specific pictures and by identifying the most significant thoughts and feelings, through six main phases: engaging in preparatory organization; reading the texts deeply; coding data; interpreting themes; searching for alternative explanations; and producing the final report (Zamperini et al., 2016, 2017).

The heart of this process was the identification of some fundamental themes, following a “bottom-up” kind of approach (that is, the themes only became clear as the analysis proceeded), that emerged from the participants’ interviews, for all the three groups. This was performed by highlighting some recurrent words or concepts that appeared to be particularly meaningful for a participant (since during the interview the person had repeated them often or had given emphasis to them), and by subsequently confronting these elements with the ones that could be found in the other participants’ narrations (Testoni et al., 2019c). The elements that appeared to be shared the most among the participants were then grouped together and formed different broader thematic categories.

Lastly, as a final step of the process, the researchers also proceeded to compare the fundamental themes discussed by each group with the ones emerged from the other two, in order to assess common elements and differences (Testoni et al., 2016).

The analysis was performed with the software Atlas.ti (Muhr, 1991), which allows researchers to directly work on written texts, highlighting portions of them, creating labels to insert in each text that can adequately represent its fundamental themes, and elaborating broader clusters of meaning comparing the data gained from each text.

The study was approved by the Ethics Committee of the Friuli Venezia Giulia Region (prot. n. 144 del 03/04/2019).

## Results

The analysis showed that each group was characterized by specific themes, which were really different, despite some similarities. The prevalent theme of the first group, still under oncological treatment, was “observing life’s equilibrium from above”; for the second group, in a follow-up phase, it was “the absence of a future”; and for the third, made of people who had never received an oncological diagnosis, “from the past, the future.”

These peculiar themes emerged explicitly from the participants’ words as general considerations, thoughts and feelings, expressed with different terms and modalities, and were then identified by the researcher who managed the data analysis, regrouped together and renamed with the expressions that can be read in the present manuscript (“observing life’s equilibrium from above,” “the absence of a future,” and “from the past, the future”), in a way that could clearly and faithfully express the fundamental elements that had emerged from the participants’ narrations.

All participants responded that they enjoyed the experience; no one suggested making any changes, and almost all expressed the wish to have further encounters.

The most preferred photos represented gears; a family (parents with two children) running in a meadow; a girl walking on railway tracks. Table 2 illustrates the frequency of choice for each cluster of photos.

**TABLE 2:** Frequency of choice for each cluster of pictures.

Participants’ group	Number of preferences for each Cluster (first choice – more than a picture possible)						Number of preferences for each Cluster (final choice – only one picture possible)					
	Cluster 1	Cluster 2	Cluster 3	Cluster 4	Cluster 5	Cluster 6	Cluster 1	Cluster 2	Cluster 3	Cluster 4	Cluster 5	Cluster 6
Group 1	4	9	6	6	1	8	1	4	3	3	0	4
Group 2	12	7	4	10	4	3	4	5	1	4	1	0
Group 3	10	5	11	12	8	13	1	0	4	5	1	5

### Main Theme of the First Group: Observing Life’s Equilibrium From Above

Participants in this group, still under oncological treatment, showed a significant preference for pictures related to medical treatment and images of change. Stefano, a 17-year-old boy with a diagnosis of an inguinal giant cell tumor, for which he faced surgery, chose the picture of a person doing a rock-climbing activity, commenting:

“In my opinion, desire consists of something people want to reach in their future. I think that the best desire is feeling good with yourself and with others. In this picture, I see a person who is climbing a mountain and who is putting a lot of effort into it in order to reach his objective. It is very similar to my brother because he wanted to enter the military. Unfortunately, he didn’t succeed because the psychologist rejected him. However, he should not give up and should try again to reach his objective. I’ve been experiencing something similar. I have to overcome difficulties, like my brother.”

Giada, a 23-year-old girl who was suffering from non-Hodgkin lymphoma, and who chose a picture representing a person climbing a mountain, said:

“A desire when you are healthy is very different compared to when you have cancer. I chose this picture that shows a person climbing a mountain because I used to do that on weekends. When you reach the top of the mountain, you see the world in front of you and feel so little. Now, I want to get back and do it again and experience the final wellbeing that the panorama always offers you. My desire is to enjoy the general things in life more. When I’m healed, I’ll live as before, but loving everything more and more.”

Luca, a 23-year-old with a diagnosis of gonad cancer undergoing surgery and chemotherapy, chose a picture representing some gears, and said:

“I chose this picture of gears because they are perfectly paired. I believe that wellbeing consists of the equilibrium derived from perfectly matching everything. However, when you are healthy, it is very difficult to understand this. However, it is impossible for everything to always go so perfectly, and it is precisely when things go wrong that you actually appreciate everything you do and have. This photo describes a perfect equilibrium between your wellbeing and the world around you.”

Similarly, Heidi, a 25-year-old undergoing chemotherapy and radiotherapy after two surgical operations because of intestinal cancer, chose a picture representing railway tracks, and explaining:

“My desire is life; it is the biggest desire I have. Living and wellbeing, compatible with my actual oncological condition. Wellbeing is to know that life is made from both happiness and pain. Indeed, all these therapies that cause so much suffering offer me the possibility to live. This prolonged illness has taught me that we must be flexible and adapt and appreciate the fact, even when they cause terrible suffering.”

Enrico, a twenty-one-year-old who had undergone chemotherapy and a surgical operation because of a medulloblastoma, chose a picture representing a desert with a man walking in the distance, leaving footprints in the sand:

“This photo reminds me of the possibility to move on, to travel not only physically but also metaphorically. It also reminds me of the importance of never forgetting loved ones and of sustaining each other. These people look ahead and never forget their past. This image gives a sense of help between people. They are people who support each other.”

Lycia, a 15-year-old girl who faced a surgical operation, chemotherapy and radiotherapy because of a bone sarcoma, chose the picture of the family running in a field because before being sick, she liked jogging:

“I wish I could be like I was before; I wish I could run again, be with my family everywhere. I love to stay with my parents and siblings and do everything with them. Now I miss them.”

### Main Theme of the Second Group: The Absence of a Future

One of the themes preferred by the second group, in a follow-up phase, belonged to the first photo cluster (i.e., leisure). 20-year-old Marco, who had been cured of Hodgkin lymphoma, expressed the desire to *“return to play sports, to get back in shape,”* choosing a picture representing a variety of sports. Conversely, Francesca, a 17-year-old girl, healed from a temporal ganglioglioma, chose a picture representing a group of healthcare professionals, however, she was extremely undecided between that and another one representing dancing, and said:

“I really love dance, and when I dance, I can express many emotions, but I am not sure what I would like to do in the future, whether to continue to dance or not. When I was sick, I discovered that thinking about becoming a nurse made me feel better.”

Giulia, an 18-year-old cured of a Ewing sarcoma 2 years before, chose a picture representing a journey, commenting:

*“A desire of mine is something I want to realize that would make me really happy. Before cancer, I liked to visit new cities so that I could discover different cultures and languages, making new friends in different contexts. Before cancer, I liked seeing new places and jumping into new adventures. This photo reminds me of all this because it is very similar to a photo I took with my mobile phone when I went to Greece*,… *with a hat and a pair of sunglasses on display.”*

Laura, a 21-year-old cured of Hodgkin lymphoma a year before, also chose the picture representing a journey, and saying:

“It gives me the idea of traveling and having new experiences. After cancer, I realized that before it, I tended to postpone everything. It is better not to delay. I don’t mean do everything immediately; however, we need to be active. The picture gives me an idea of wellbeing as light-heartedness, freedom and peace of mind.”

Paola, a 25-year-old, who had Hodgkin lymphoma up to 4 months earlier, chose a picture representing a tightrope walker, and referring to her situation:

“My present desire is to have some peace, to live my life fully without having to think about other things that constantly jeopardize my wellbeing. I like this photo because it really expresses my current feeling since this person is actually walking on a wire. It is an extreme sense of uncertainty with respect to the future.”

Maria, a 15-year-old girl who had overcome Hodgkin lymphoma a year before, chose the picture of the family running in a field, and saying:

“This picture makes me think of the wellbeing of a family. I want my family to be all right, and I don’t want those I love to have thoughts that could be too painful about me. This is my desire; that my family could indeed be happy.”

Elisa, an 18-year-old, healed of Hodgkin lymphoma 8 months before, chose a picture representing some colorful crayons, commenting:

“I want to color my life a little more now. I wish to engage in more activities. Especially during summer when I have more time and I do not have to go to school, I would like to travel. The meaning of this picture is to make life colorful.”

The theme most preferred, however, was that of the second photo cluster, and Camilla, a 21-year-old, who had been affected 27 months earlier by non-Hodgkin lymphoma, perfectly represented the narrations of other participants of this group. She chose a picture illustrating a blank piece of paper, saying:

“This is my desire. It represents something that you strive to reach. This white describes everything you need in life. Something temporary, that is, which is constantly evolving. It seems that you can write your life as you want on the white paper. Actually, I am still trying to decide what my specific desires are, and this picture represents this emotion.”

### Main Theme of the Third Group: From the Past, the Future

Participants of the third group, who had never experienced a serious pathology, chose mainly photos related to “family life” and “change.” Matteo, a 17-year-old boy who chose a photo representing a family, explained:

“My desire would be to study dramatic arts. This is my true passion. However, I understand that it is difficult to find a job in this field, and this could make it difficult for me to start a family. Indeed, what I feel more urgently now is starting a family. I was an orphan, so I know how important it is to have a family where you are loved. Living without parents was very difficult, even though I have always had many people surrounding me who offered me their support. This photo reminds me of a picnic I had with my grandparents. I remember I came back home that evening and before going to bed, I cried because I was happy.”

Elisabetta, a 17-year-old, also chose the picture of a family:

“I believe a desire can be a specific expectation or something a person strongly wants to reach. There are many pieces that compose my life, and I am trying to complete them all. I prefer to choose the few people I can trust to talk to about myself and spend time with to deepen the relationship. I love my sister very much, but she got married. I miss her, but she has two little children, and it is as if they are another piece of my life. And with them, I have discovered another kind of intimacy, a new part of me, as if another piece of the puzzle has arrived. I hope to have a family of my own soon.”

Sofia, a 19-year-old, chose the picture of rock climbing, explaining:

“Desire refers to something I don’t have right now; it’s something I aspire to for the future. It is a way to set a goal. Something to achieve. When I have a desire, I do everything in my power to realize it. I want to do everything in my power now because one day I will not be able to do it anymore. In fact, there are desires that you cannot realize anymore, especially when you meet death. A few months ago, the mother of a friend of mine died. I had never visited her; I kept postponing this duty because I would not have been able to say anything. When she died, it was traumatic, and this made me want to do everything, even though it pushes me out of my comfort-zone.”

Similarly, 19-year-old Massimo chose the picture of a girl walking on some railway tracks and described how desire develops in a negative context:

“This girl is trying to go down her road, but she has the train behind her, which is almost crushing her or is making her run and is leading her in a certain direction. She cannot walk peacefully in this environment. However, this picture gives me courage because she is trying to do what she desires serenely without worrying about the context. The context is negative, but the girl’s reaction is positive, because she does not see the train or is ignoring it, or she has decided to keep going anyway.”

## Discussion

### Main Themes Emerged Within the Three Groups

This study confirmed that PhotoTherapy can help adolescents and young adults manage difficulties related to severe illnesses and negative past experiences by allowing them to reflect upon their present condition between the past and the future. The groups of themes that were most popular were as follows: changes (first and third groups, that is, participants with a current oncological diagnosis and people who never had one in their life), in particular the images of climbing mountains; medical treatment (first and second groups, that is, once again participants with a current oncological diagnosis and the ones who had a past one), often images of physicians and surgeries; family (third group, made of people who had never experienced cancer), and especially people at the table. The most important themes that emerged in the narratives of the first group (oncological patients) were related to the difficulties caused by illness and the desire to have the strength to face the fatigue of the climb that the disease entails, to finally be able to look at the panorama of life and its equilibrium from above, finally reaching wellbeing. On the contrary, the main representation that emerged from the narrative of the second group (former oncological patients) describes the present, with an intense need for the restoration of wellbeing, while the third group (people who had never experienced cancer) considered it something that may be put aside.

Some important differences between participants in the first (oncological patients) and third (people who had never experienced cancer) groups also emerged with regard to the idea of change. In fact, the latter interpreted it as wellbeing that had developed from the maturation process, whereas the first group interpreted it as its restoration. In the first group, the desired change was related to past passions that cancer had blocked, as described by Giada and Luca, who were reminded of their previous condition and considered the present as a transitory situation. In contrast, the third group, made of people who had never been ill, desired future perspectives that involved friendship and the construction of family, as narrated by Camilla and Matteo. Furthermore, among these last participants, someone found the pictures to serve as a starting point from which to spontaneously explore themes of death and vulnerability. For example, Sofia expressed her regret for not having had the courage, in the past, to visit the terminally ill mother of a friend of hers.

### Peculiarities of the Second Group

Particular attention should be paid to participants in the second group, that is, people who had a past oncological history, now in a follow-up phase, who presented some peculiarities. Those participants preferred themes related to the process of treatment, and the image of a white piece of paper described by Camilla excellently represented their particular condition of uncertainty with respect to the future. Furthermore, in contrast to participants in other groups, who described expectancies related to health restoration or to the classic developmental tasks of their age, the second group chose pictures representing neither “change” nor “school and job.” On the one hand, they mainly expressed the hope that everything would remain stable for their present health condition, and on the other, they mentioned a void with respect to future aims. This condition was clearly depicted by Paola, who compared her situation to that of a tightrope walker, on the edge between health and illness, between the will to move forward and the risk of not being able to do so.

Examining the differences in the representations of the family, we can suggest a connection between the third and first groups (that is, participants who had never experienced cancer and who had a current oncological diagnosis, respectively) in order to understand what could have happened in the second group. The desire to establish their own family was represented by participants in the third group as a way of building their own future. In contrast, the first group paid particular attention to and demonstrated a great sense of responsibility with regard to their natural family. This was related, as Enrico described, to the need to be close to those he loved, not only to receive support from them but also to give it back in a mutual act of love. In the second group (made of people who had received an oncological diagnosis in the past and completed their treatment), this characteristic reached its maximum expression, as Maria made clear when describing her concerns with respect to her family’s wellbeing. Indeed, she expressed her intense desire not to be a burden for those she loved. In this horizon of insecurity, the most relevant desire was the restoration of wellbeing through leisure because it made it possible not to think too much about past suffering, duties and sense of responsibility toward others.

### The Impact of an Oncological Pathology on Young Patients and Future Perspectives

All this indicates that the end of the pathological condition does not correspond to the resumption of psychological wellbeing. As indicated by the literature (Smith et al., 1991; Grinyer, 2007; Sawyer et al., 2017), cancer has a very significant psychological impact on young people, even when the pathology has been overcome. Our research confirmed that the actual impact of threatening illness on people this age is particularly grave (Grinyer, 2007) because the negative psychological and social consequences may last for a long time even after the disease has been cured (Smith et al., 1991).

Finally, some significant information was also offered by the pictures that were not chosen. For example, the cluster related to personal identity was neglected by all groups, and absolutely so by people who were currently under oncological treatment (first group). This result could be interpreted as the effect of an intense uncertainty concerning the perception of the somatic dimension, which for young people is constantly evolving but which is frustrated by the experience of cancer (Tindle et al., 2009; Lee et al., 2012). This particularity suggests that the emergence of this pathology blocks any further expression of desire with respect to the body, which is entirely exemplified by the words for physical health losing both their aesthetic and erotic characterization.

Furthermore, on the one hand, the third group, that is, participants who had never experienced cancer, avoided speaking about the wish to remain sane, but on the other hand, they spontaneously considered the issue of death and loss, saying that they had not had the opportunity to speak of these experiences before. All this allows us to consider the fact that there is a lack of educative reflection on these themes at school, as is widely considered by the literature on death education, and confirms that the use of photos can significantly help to facilitate this matter (Testoni et al., 2018b, 2019a).

Our study confirms that PhotoTherapy can be considered a useful methodology that facilitates discussion concerning delicate and potentially distressing issues, as some other researchers have previously highlighted (Weiser, 1999; Musacchi, 2016; Gaggiotti et al., 2019). It proves to be a particularly useful tool for therapists, especially when dealing with seriously ill adolescents or young adults whose wellbeing represents a complex challenge for healthcare providers (Wiener et al., 2015).

## Conclusion

The research has highlighted that PhotoTherapy can support seriously ill adolescents and young adults suffering from the psychological consequences of cancer by facilitating the expression of their desires, hopes and fears and enhancing their wish to have their wellbeing restored. Indeed, the expression of satisfaction regarding the experience confirmed how positive it was precisely because it allowed everyone to address significant existential issues, such as sickness, loss or being an orphan.

However, the study dramatically confirmed that cancer has an intense impact on young people, negatively affecting the way they imagine their future, their desires and needs, and the way they approach wellbeing. In particular, the research highlighted that during the illness, the main desire is healing, and this need has the power to erase all other desires beyond a sense of gratitude and responsibility toward the natural family.

PhotoTherapy could serve as a useful tool to support the recovery of images that ignite desires and needs in young people, to fully reactivate their strength to imagine a future and to motivate their achievement of the next developmental steps. Indeed, this technique is able to help suffering people reflect upon existential issues in a condition of emotional safety, thus offering them an occasion for a proper reactivation of their aspirations to wellbeing.

The limits of this study are related to the small number of participants. Further studies could consider more specifically the different psychological conditions crossing the pathology and the iatrogenic effects of the active cures with time and age, defining the effect of PhotoTherapy on more specific groups of participants. Furthermore, it would be very important to utilize all the forms of intervention described by Judy Weiser and to survey their different effects.

## Data Availability Statement

All the materials used to conduct the present research can be made available upon request to interested researchers. The raw data supporting the conclusions of this article will be made available by the authors, without undue reservation, to any qualified researcher.

## Ethics Statement

The studies involving human participants were reviewed and approved by Ethics Committee of the Friuli Venezia Giulia Region (prot. n. 144 del 03/04/2019). Written informed consent to participate in this study was provided by the participants’ legal guardian/next of kin.

## Author Contributions

IT is the scientific director of the present research project, she therefore elaborated the study design and supervised its implementation. She also contributed in the interpretation of the data and the recognition of the mains results, supervised the elaboration of the manuscript, and wrote its final version. ET was the researcher directly responsible for the data collection and its analysis. She therefore encountered the participants, conducted the PhotoTherapy sessions and the interviews and later analyzed the obtained data, producing the results that are described in the present manuscript. She also recruited the participants of the control group. SP wrote the first draft of the present manuscript and she also translated the manuscript and the quotations cited in the Results section in English. She also handled the editing process of the manuscript. MM is the Doctor responsible for the Young Area inside the Oncological Reference Center of Aviano (Italy), so he organized with the structure all the procedures to allow the implementation of the research there and he also handled the recruitment of the participants in the first two groups (that is, adolescents and young adults who had at the time of the research or had had in the past a diagnosis of cancer, since they were all treated, or had been treated inside the Aviano Centre). MW offered suggestions for the enhancement of the arts therapy intervention and participated in the discussion of the results.

## Conflict of Interest

The authors declare that the research was conducted in the absence of any commercial or financial relationships that could be construed as a potential conflict of interest.
