# Supplementary material for: Can Desire and Wellbeing Be Promoted in Adolescents and Young Adults Affected by Cancer? PhotoTherapy as a Mirror That Increases Resilience
Source: Front Psychol. 2020 May 12;11:966. doi: 10.3389/fpsyg.2020.00966 (PMC7235290; doi:10.3389/fpsyg.2020.00966)

Supplementary Material

Pictures chosen by the participants quoted in the manuscript

“Man climbing a mountain”. Picture chosen by Stefano, Giada (group 1, under oncological treatment), and Sofia (group 3, no experience with cancer).

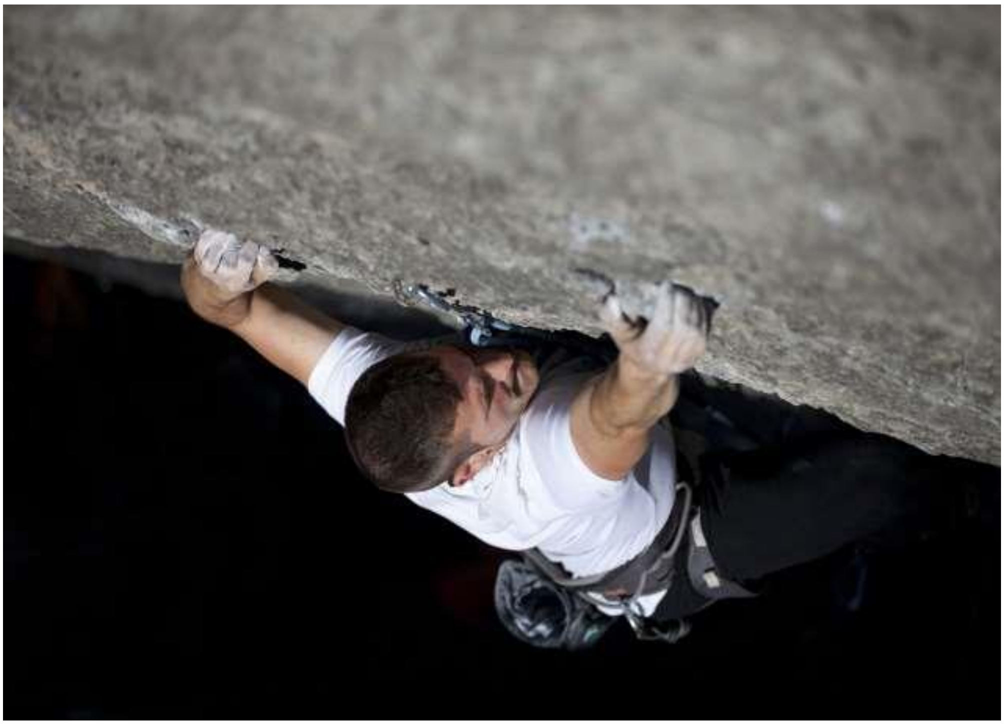

“Gears”. Picture chosen by Luca (group 1, under oncological treatment).

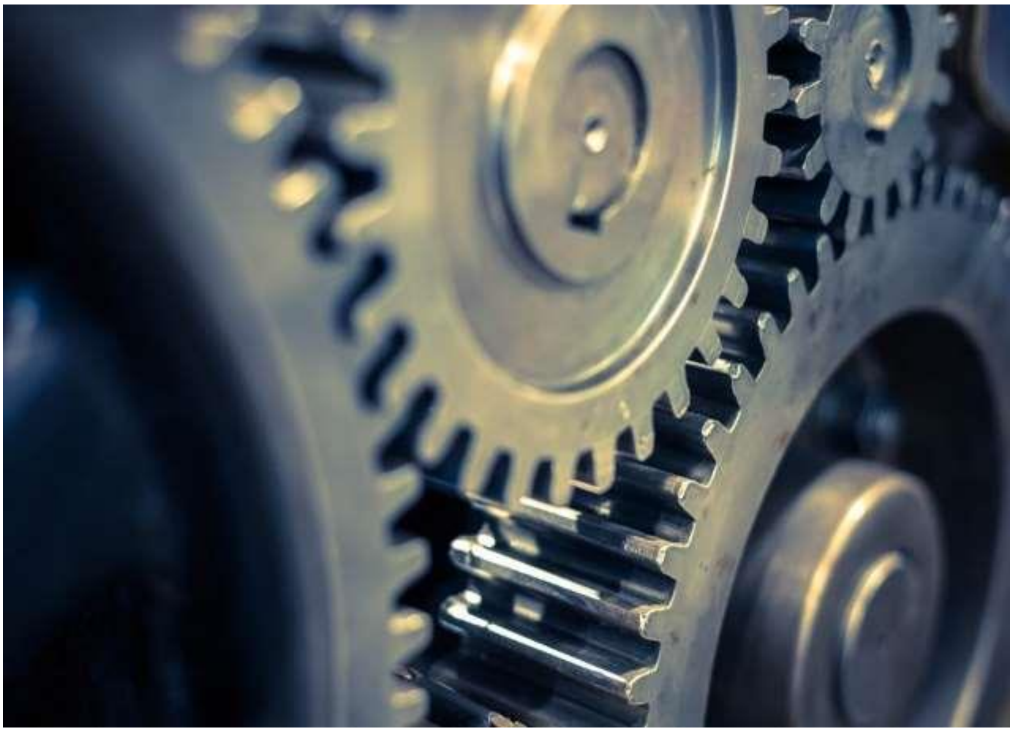

"Railway tracks with a girl walking on them". Picture chosen by Heidi (group 1, under oncological treatment), and Massimo (group 3, no experience with cancer).

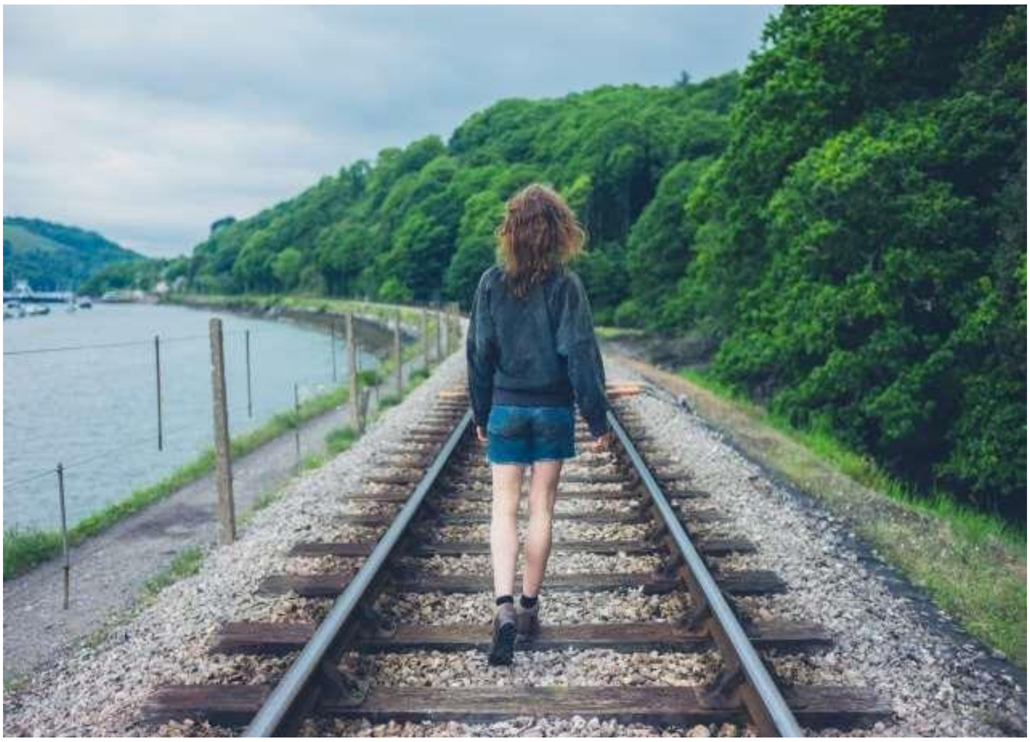

“Desert with a man walking in the distance”. Picture chosen by Enrico (group 1, under oncological treatment).

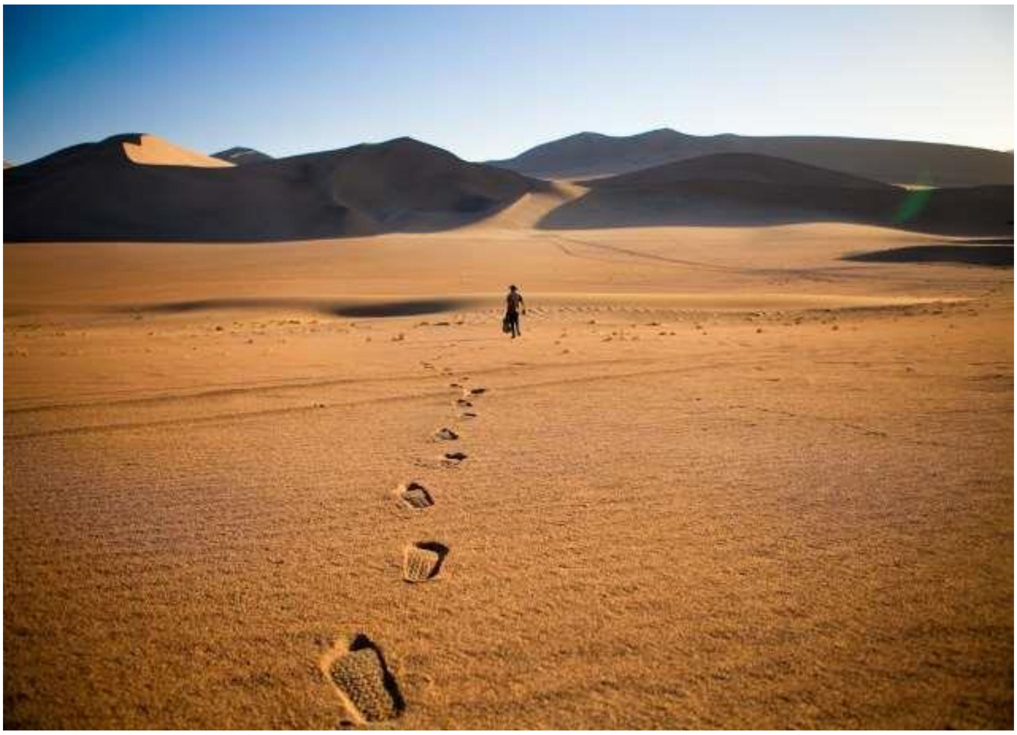

“Family happily running on a field”. Picture chosen by Lycia (group 1, under oncological treatment), Maria (group 2, in a follow-up phase after an oncological pathology), Matteo and Elisabetta (group 3, no experience with cancer).

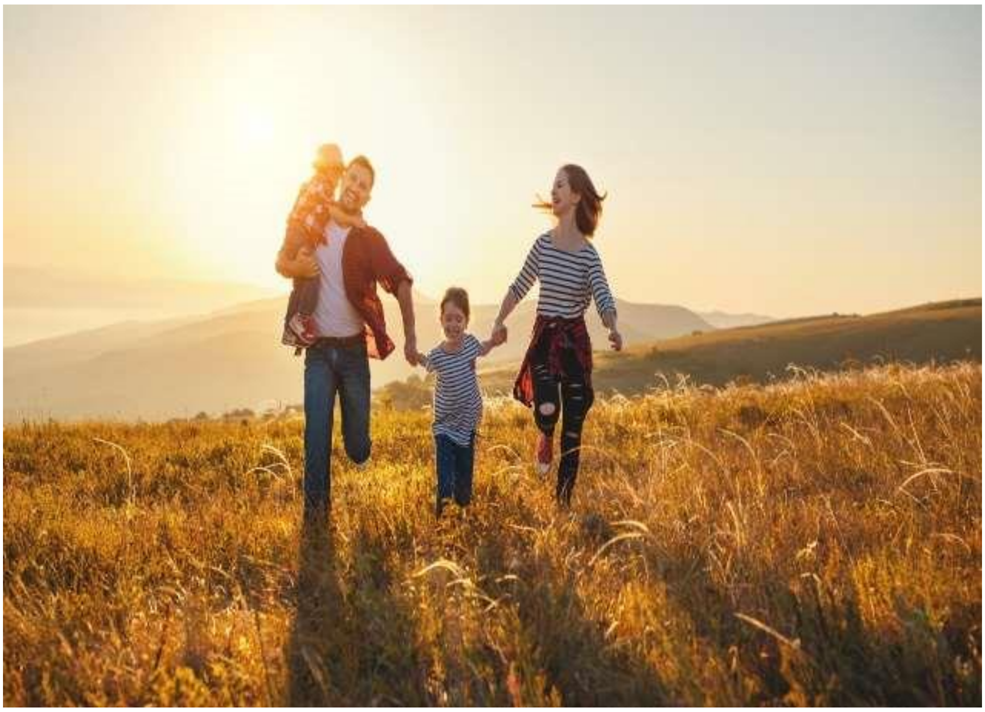

“Sports”. Picture chosen by Marco (group 2, in a follow-up phase after an oncological pathology).

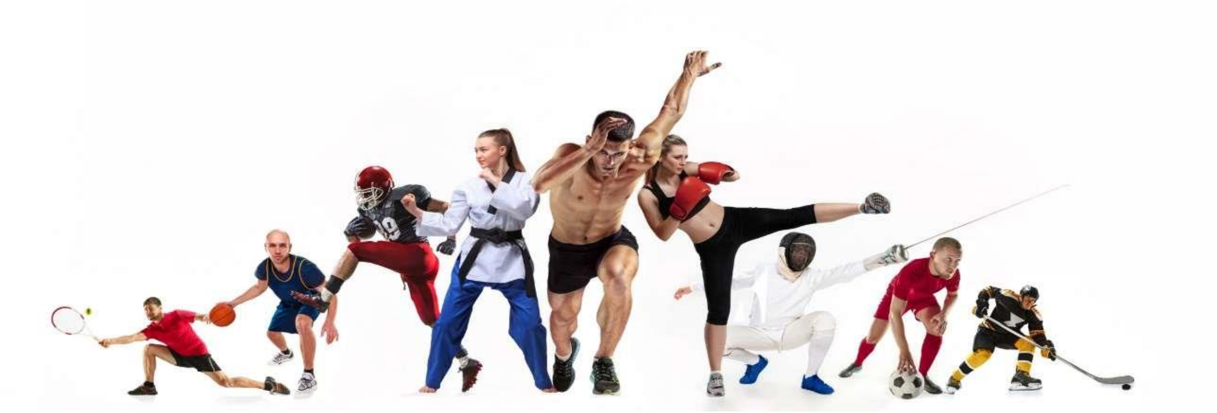

“Healthcare team”. Picture chosen by Francesca (group 2, in a follow-up phase after an oncological pathology).

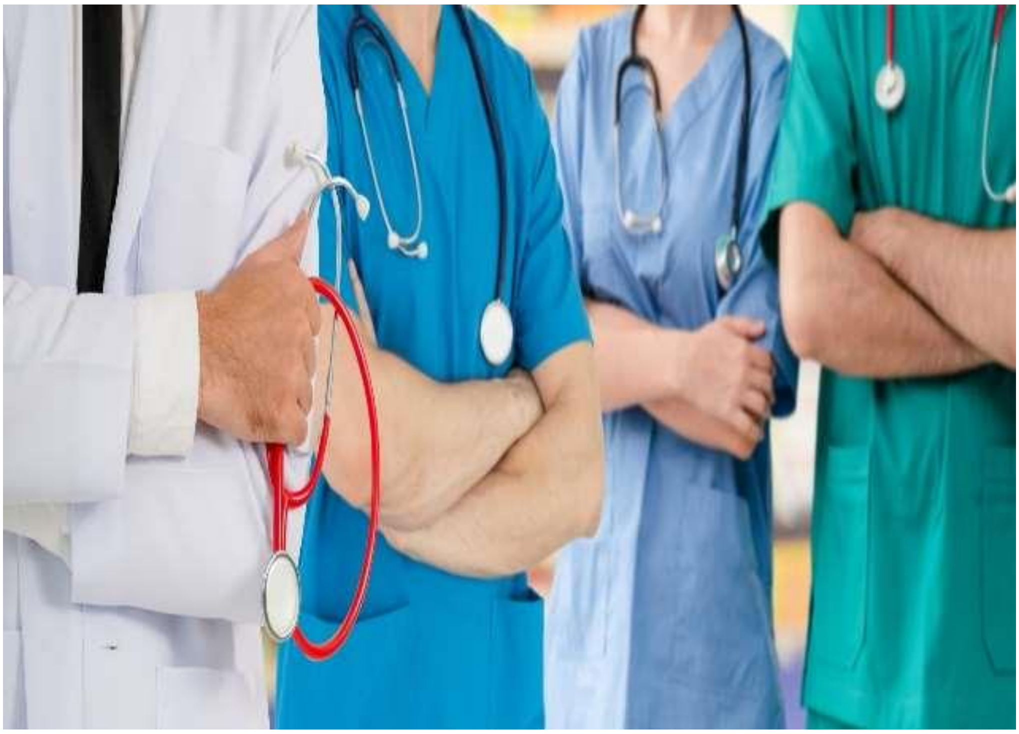

"Journey". Picture chosen by Giulia and Laura (group 2, in a follow-up phase after an oncological pathology).

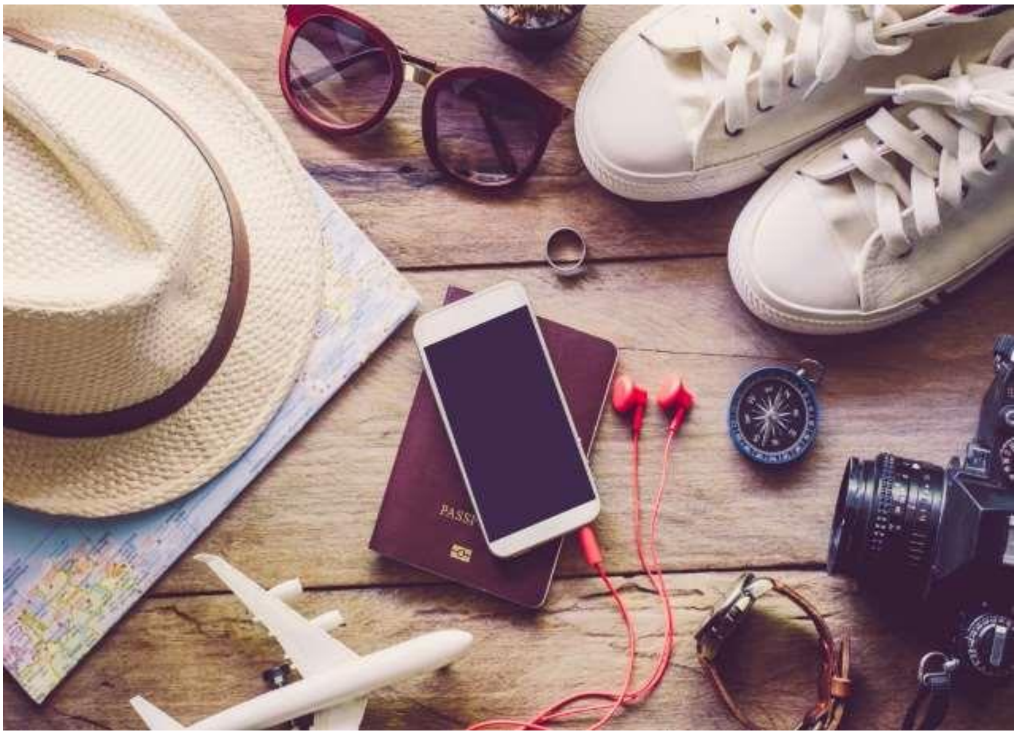

"Tightrope walker". Picture chosen by Paola (group 2, in a follow-up phase after an oncological pathology).

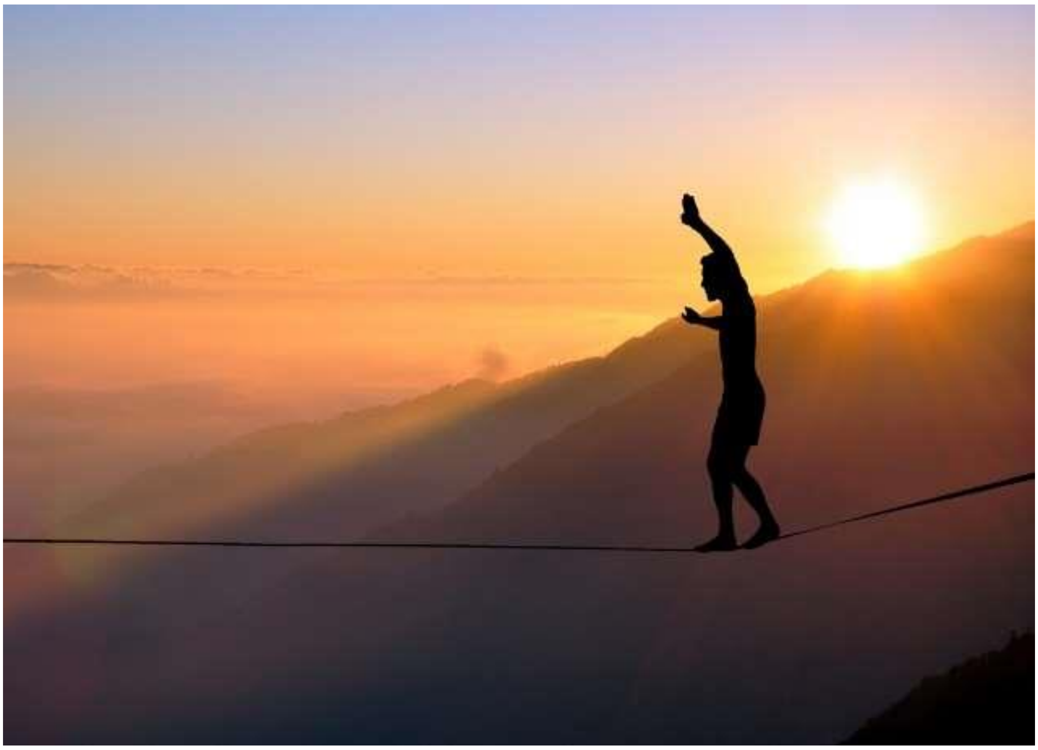

“Colorful crayons”. Picture chosen by Elisa (group 2, in a follow-up phase after an oncological pathology).

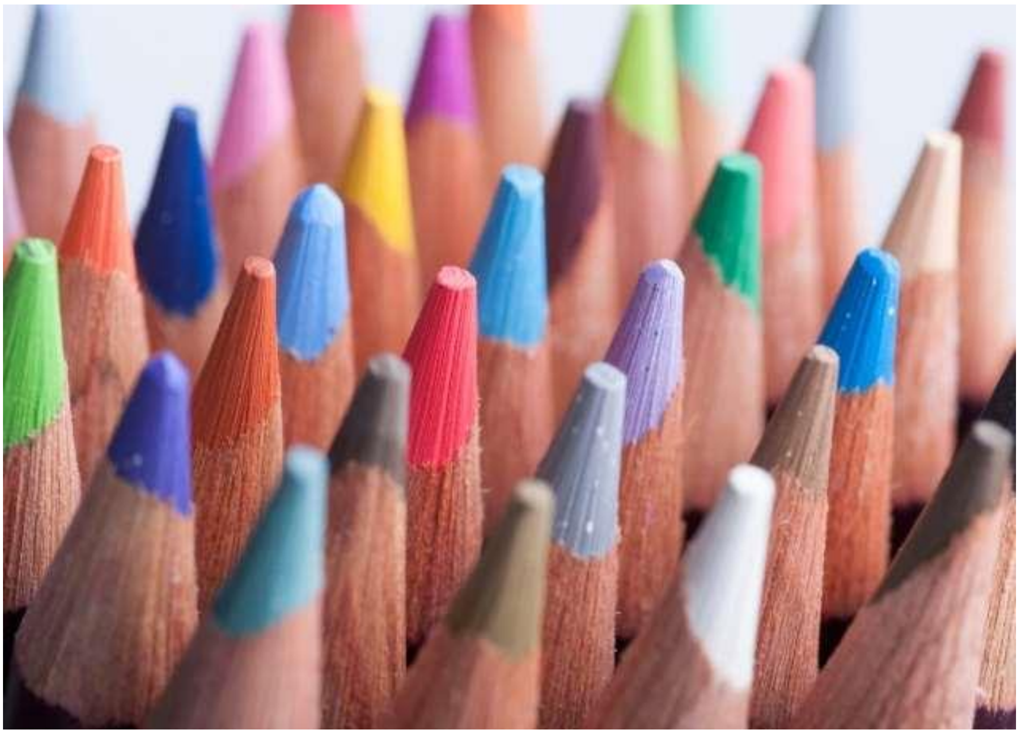

“Blank piece of paper”. Picture chosen by Camilla (group 2, in a follow-up phase after an oncological pathology).

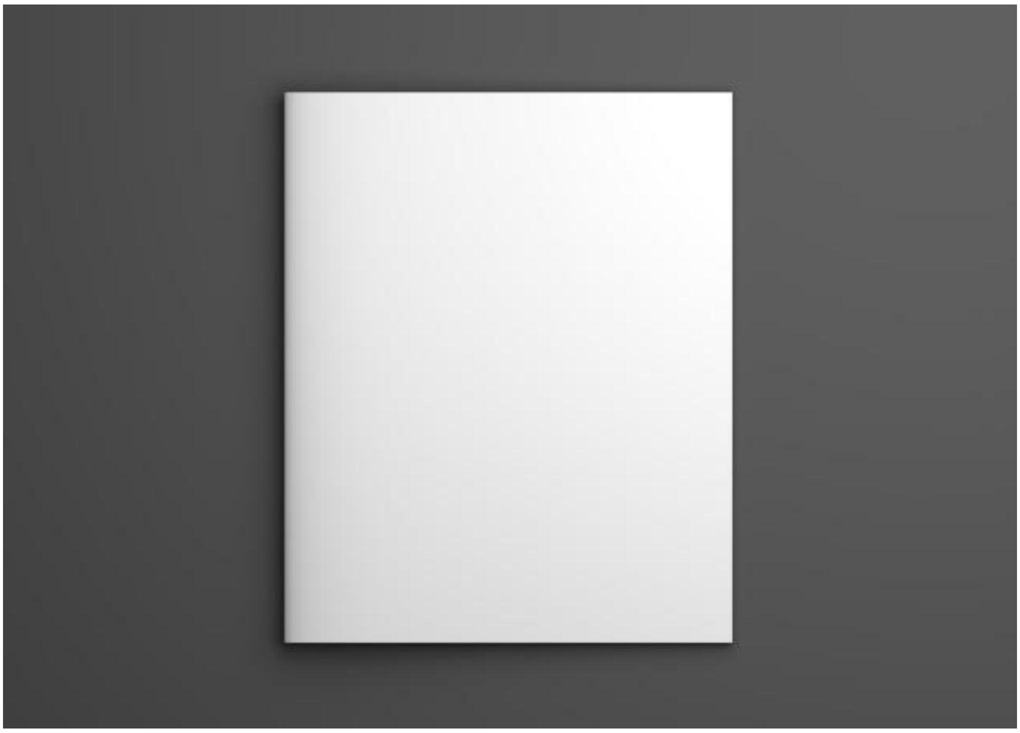

Supplement: Supplementary file 1 [file Data_Sheet_1.pdf]
